# Serology based disease status of Pakistani population infected with Hepatitis B virus

**DOI:** 10.1186/1471-2334-7-64

**Published:** 2007-06-27

**Authors:** Muhammad Masroor Alam, Soahil Zahoor Zaidi, Salman Akbar Malik, Asif Naeem, Shahzad Shaukat, Salmaan Sharif, Mehar Angez, Anis Khan, Javed Aslam Butt

**Affiliations:** 1 Department of Virology, National Institute of Health, Islamabad, Pakistan; 2 Head of Department of Virology, Principal Investigator-WHO Regional Reference Laboratory for Polio Eradication Initiative, National Institute of Health, Islamabad, Pakistan; 3 Head of Department of Biochemistry, Quaid-i-Azam University, Islamabad, Pakistan; 4 Head of Department of Gastroenterology, Pakistan Institute of Medical Sciences, Islamabad, Pakistan

## Abstract

**Background:**

The infection rate of hepatitis B virus is continuously increasing in Pakistan. Therefore, a comprehensive study of epidemiological data is the need of time.

**Methods:**

A total of 1300 individuals were screened for HBV infection markers including HBsAg, anti-HBsAg, HBeAg and anti-HBcAg. The association of these disease indicators was compared with patients' epidemiological characteristics like age, socio-economic status and residential area to analyze and find out the possible correlation among these variables and the patients disease status.

**Results:**

52 (4%) individuals were found positive for HBsAg with mean age 23.5 ± 3.7 years. 9.30%, 33.47% and 12% individuals had HBeAg, antibodies for HBsAg, and antibodies for HBcAg respectively. HBsAg seropositivity rate was significantly associated (*p *= 0.03) with the residing locality indicating high infection in rural areas. Antibodies titer against HBsAg decreased with the increasing age reflecting an inverse correlation.

**Conclusion:**

Our results indicate high prevalence rate of Hepatitis B virus infection and nationwide vaccination campaigns along with public awareness and educational programs are needed to be practiced urgently.

## Background

Hepatitis B virus (HBV) infection is a major health problem leading to significant morbidity and mortality worldwide especially in the developing countries like Pakistan. Approximately 2 billion people in the world have been infected by HBV [1], 400 million of who are chronic carriers [2]. The virus causes acute hepatitis of varying severity [3] and persists in 95% of children and 2–10 % of adult patients [4] leading to chronic liver disease, cirrhosis, hepatocellular carcinoma [5] and even fulminant hepatitis [6].

In Pakistan, HBV infection rate is increasing day by day. The reason may be the lack of proper health facilities or poor economical status and less public awareness about the transmission of major communicable diseases like hepatitis B, hepatitis C and Human Immunodeficiency syndrome.

This research study was conducted to assess the major epidemiological factors linked with hepatitis B virus infection. Therefore, serological testing of randomly selected individuals was performed for HBV infection markers and the association of individuals' demographics with the status of Hepatitis B virus infection was determined.

## Methods

### Population data

This study was completed during January 2005 to January 2006 at Serology Laboratories, Department of Virology, National Institute of Health (NIH), Islamabad after receiving approval from the Research committee of NIH.

The study included 1300 individuals including males and females, aged 8–53 years from different localities of the country. Individual's epidemiological and demographic data like age, residential area and socio-economic status was recorded on a standard designed questionnaire.

The socio-economic status was mainly assessed as two categories: poor and rich. The individuals with monthly income between Rs.5, 000 – 10, 000 and more than 10,000 were considered as poor and rich respectively. Regarding area, all the peri-urban localities, villages and small towns were included in rural area whereas the population from the main central areas of city was included to fulfill the criteria of urban category.

### Serological testing

Blood sample was taken from all individuals after getting verbal as well as written consent. 8 cc of venous blood was collected in a sterile vaccutainer and was referred to Serology laboratories, Department of Virology, NIH where centrifugation was done to separate sera. Sera were stored at -20°C until further processing. The individuals were referred for serological testing of HBV markers including HBsAg, anti-HBsAg, HBeAg and anti-HBcAg. Accordingly, each individual was tested using AxSym HBsAg MEIA, AxSym AUSAB, Abbott Laboratories, IL, USA and AxSym CORE, Abbott Laboratories, USA.

### Statistical analysis

Logistic regression was used to check out the relationship between HBV markers and associated risk factors. A p-value ≤ 0.05 was considered as statistically significant.

## Results

**Figure 1:**
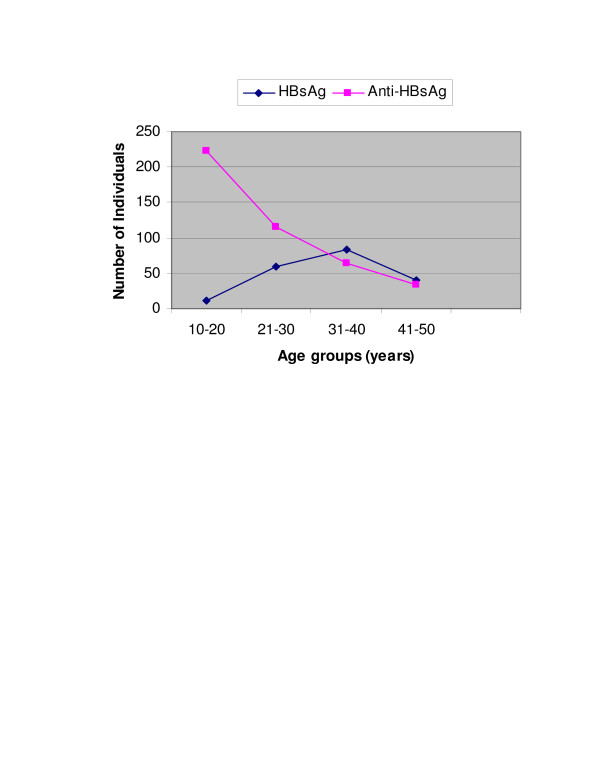
Frequency of HBsAg and Anti-HBsAg seropositivity among various age groups.

Out of the total 1300 individuals screened, 52 (4%) were positive for HBsAg. Males were found to be more frequently positive for HBsAg than females (64% vs. 36%). The mean age of individuals positive for HBsAg was found to be 23.5 years (± 3.7). The frequency of HBV infection was found to be higher in the individuals aged 30–40 years while anti-HBsAg level was higher in younger individuals and level descended with the increasing age as shown in Figure 1. 435 (33.47%) individuals were found with anti-HBsAg above the detection limits including 23.14% males and 10.33% females. The individual positive for HBsAg were retested for confirmation and then tested for presence of HBeAg. 121 (9.30%) of them were found positive for HBeAg. Antibodies to core antigen were found in 156 (12%) of the total individuals enrolled. Twenty four of them were also positive for HBsAg.

The regression analysis was performed to assess the relation of HBV disease status with the patients epidemiological characteristics which showed that HBsAg seropositivity was significantly associated with residential area and socio-economic status of individuals showing rural locality and poverty as the major risk factors involved (*p *= 0.03 and *p *= 0.04 respectively). It was analyzed that rural population is at 3.8 fold high risk of HBV infection as compared to those residing in the urban localities. Similarly, poverty is also linked with high HBV infection rate posing the poor population at a 2.2 fold high risk of infection. Regarding age, the individuals aged 21–30 years of age were found to be at 2.7 times higher risk of HBV acquisition followed by 31–60 years of age period vulnerable for infection (Table 1).

**Table 1:** Multiple Regression analysis representing the association of individuals' variables with the seroprevalence of Hepatitis B virus

**Risk factors**	**HBsAg **+ (Out of 1300)	**OR (95% CI)**
**Residential area**		
Urban	14	
Rural	38	3.8 (1.6–9.8)
		
**Socio-economic status**		
Rich	19	
Poor	33	2.2 (1.3–3.7)
		
**Age (years)**		
<20	06	
21–30	13	2.7 (1.6–4.9)
31–40	21	1.2 (0.7–2)
41–60	10	1.2 (0.9–3.2)
>60	02	0.8 (0.5–1.2)

## Discussion

In Pakistan, a large number of studies have been carried out regarding HBV prevalence rate and epidemiological issues. All such studies present a quite variable picture of the disease depending on the factors focused like sample size, objectives of study, associated risk factors, population under study, diagnostic assays practiced, ethnicity, socio-economic status and general population behavior.

The present study is based on Pakistani population selected on random basis including individuals from all the four provinces of Pakistan. Almost all of the previous reports showing the country disease picture are based on the patients' data that visited hospitals or were found to be clinically affected. Furthermore, a major part of such studies are based on selected groups known to be highly vulnerable for the disease acquisition. Therefore, it was the need of time to explore the current figure of infected population.

According to WHO, Pakistan falls in the low endemic area of HBV infection with prevalence of 3% infected population. Hussain *et al*., in 1998 [7] reported 7.8% incidence of HBV infection with male to female ratio of 7:1. A study from Bahawalpur showed 2.9% prevalence of HBV carriers among local population [8]. A study conducted at Armed Force Institute of Transfusion, Islamabad showed that 3.3% blood donors from Northern Pakistan were HBsAg positive [9]. HBV prevalence was found to be 2.04% in Lahore while 2.06% in healthy blood donors of Faisalabad [10] The frequency of hepatitis B antigen and antibody determined in healthy subjects and patients with liver disease was 2.9% and 35% respectively while 33% patients with acute viral hepatitis, 20% with cirrhosis and 10% with hepatocellular carcinoma (HCC) were HBsAg positive [11]. HBV prevalence rate of 2% had been reported in the male volunteer blood donors of Karachi [12]. 55% of the chronic liver disease and hepatocellular carcinoma patients were positive for HBsAg [13]. The seroprevalence of HBsAg in male sex workers at Karachi showed positivity rate of 3.4% [14].

In our study, most of the individuals positive for HBsAg and HBeAg were belonging to low class socio-economic status and rural areas. Pakistan lies between middle to low income countries with over one-twelfth of labor force unemployed, where over one fifth of the population subsides in poverty and over half of the population is illiterate [15]. According to Population Census Organization, Pakistan has about 165.80 million population with 67.5% living in rural areas while urban population is comprised of 32.5% of total population (16). It has been well documented that HBV infection is more prevalent in low socio-economic settings in majority of the world regions like Indonesia [17] and similarly in Pakistan.

The high level of anti-HBsAg in the younger age is an indicative of the progressive efforts of EPI after inclusion of HBV vaccination in the routine immunization practice. Public awareness plays a much important role in the prevention and control of infections especially those having no proper or specific treatment and cure. Good management practices are proven to be the gold standard ways in order to get control of such dead-end diseases like HBV, HCV and HIV.

There were only 16 (1.23%) patients found to be vaccinated against HBsAg. Further more, almost all of the other patients declared that none was either vaccinated against HBV, representing very little vaccination coverage. Unawareness and Cost effectiveness were found to be the main issues regarding very little vaccination coverage.

The risk factor involved in the recent outbreak of HBV infection reported from Larkana (Sindh province of Pakistan) has been found to be the intravenous drug usage. The limitations of our study include the lack of information about HBV associated risk factors like multiple blood transfusions, surgical operations especially haemodialysis, dental procedures, unsafe sex practices, frequent barber visiting and horizontal transmission modes. Also, the target populations like intravenous drug users and addicted population are more prone and vulnerable to get and transmit infection frequently and needs particular attention.

## Conclusion

Hepatitis B virus infection is widespread in Pakistan and has led to a higher incidence of acute and chronic liver diseases in the region. Establishment of defense measures against hepatitis virus infections is an important and urgent matter for public health. It is imperative that for eradication of HBV infection, universal vaccination of all new born is carried out together with education of the public to limit the transmission of HBV infection to those who are safe and free of infection. In 2001–2002, Pakistan received a grant from the Global Alliance for Vaccines and Immunization (GAVI) that has enabled the introduction of Hepatitis B vaccination in routine Expanded Program on Immunization (EPI) [18]. Vaccination for HBV as a part of EPI was launched in a nationwide vaccination campaign in 2004 [19]. Special attention was given to children under 1 year of age. Another encouraging effort towards the development of a better and healthier society is the initiation and implementation of "Prime Minister Program for the prevention and control of Hepatitis" since the mid of 2006. The disease scenario is hoped to be changed since the awareness campaigns initiated by the Ministry of Health and other local bodies struggling for the well being and good health of the society.

## Competing interests

The author(s) declare that they have no competing interests.

## Authors' contributions

SSZ and SAM designed the Research project and gave a critical view of manuscript writing, JAB helped in providing the samples, MMA, AK and SS collected the epidemiological data, AN analyzed thee data statistically, SS and MA performed the serological assays and MMA wrote the manuscript. All the authors have read and approved the final manuscript.

## Pre-publication history

The pre-publication history for this paper can be accessed here:


